# Cost of adapting the International Guide for Monitoring Child Development, Guatemala and India

**DOI:** 10.2471/BLT.25.294361

**Published:** 2026-04-02

**Authors:** Scott Tschida, Anuj Mundra, Revan Mustafa, Amruta Bandal, Magdalena Guarchaj, Karyn Choy, Priyamvada Das, Sara Hernandez, Ashwini Kalantri, Abhishek V Raut, Anushree Sane, Roopa Srinivasan, Vibha Krishnamurthy, Ilgi Ertem, Subodh S Gupta, Peter Rohloff, Chunling Lu

**Affiliations:** a Center for Indigenous Health Research, Maya Health Alliance, Tecpán, Guatemala.; b Mahatma Gandhi Institute of Medical Sciences, Sewagram, Maharashtra, India.; c Department of Pediatrics, Acibadem Maslak Private Hospital, Istanbul, Türkiye.; d Ummeed Child Development Centre, Mumbai, Maharashtra, India.; e Department of Pediatrics, Faculty of Medicine, Ankara University, Ankara, Türkiye.; f Division of Global Health Equity, Brigham & Women’s Hospital, Department of Global Health and Social Medicine, Harvard Medical School, 641 Huntington Avenue, Boston, MA 02115, United States of America.

## Abstract

**Objective:**

To assess the cost of co-creating an adaptation of the International Guide for Monitoring Child Development (intervention for use by community health workers (CHWs)) in rural Guatemala and India.

**Methods:**

We developed survey instruments to capture the costs, from health-system and societal perspectives, of adapting the guide with local partners and international experts in the two sites. We included the costs of human resources, information technology, and infrastructure and logistic support. To improve the transparency and comparability of cost estimates, we reported the required resources for adaptation, such as time and expertise of human resources, and the quantity, function and usage time of the resources used.

**Findings:**

Adaptation of the guide took 14 months in India and 18 months in Guatemala during 2021–2022. Total costs by site were 38 174.57 United States dollars (US$) in Guatemala, US$ 39 287.15 in India and US$ 81 846.59 for international consultants. International consultants accounted for about half of the costs, and the Guatemala and India sites each accounted for about a quarter of the costs. Human resources were the largest contributor to the adaptation costs in both sites (90.1%; 34 398.20/38 174.57 in Guatemala and 94.8%; 37 262.74/39 287.15 in India) followed by infrastructure support and information technology.

**Conclusion:**

Since most of the required steps to adapt the guide for use by CHWs have now been done by our study, we expect future adaptation costs to be lower. The methods used in this study provide an example of how to cost intervention adaptations in the future.

## Résumé

**Objectif:**

Évaluer le coût de la création conjointe d'une adaptation du Guide international d’observation du développement de l’enfant (intervention destinée aux agents de santé communautaire) dans les zones rurales du Guatemala et de l’Inde.

**Méthodes:**

Nous avons mis au point des outils d’enquête afin de recenser les coûts, d’un point de vue sociétal et du système de santé, liés à l’adaptation du guide en collaboration avec des partenaires locaux et des experts internationaux dans les deux pays. Nous avons pris en compte le coût des ressources humaines, de l’équipement informatique, de l’infrastructure et du soutien logistique. Afin d’améliorer la transparence et la comparabilité des estimations de coûts, nous avons énuméré les ressources nécessaires à l’adaptation, telles que le temps mis et l’expertise des ressources humaines, ainsi que la quantité, la fonction et la durée d’utilisation des ressources mobilisées.

**Résultats:**

L’adaptation du guide a nécessité 14 mois en Inde et 18 mois au Guatemala sur la période 2021–2022. Les coûts totaux par pays étaient de 38 174,57 dollars américains (USD) au Guatemala, de 39 287,15 USD en Inde et de 81 846,59 USD pour les consultants internationaux. Ceux-ci représentaient environ la moitié des coûts, tandis que l’Inde et le Guatemala représentaient chacun environ un quart des coûts. Les ressources humaines représentaient le poste de dépenses principal dans les coûts d’adaptation dans les deux pays (90,1%; 34 398,20 / 38 174,57 au Guatemala et 94,8%; 37 262,74 / 39 287,15 en Inde), suivies de l’infrastructure et de l’équipement informatique.

**Conclusion:**

Dans la mesure où la plupart des étapes nécessaires à l’adaptation du guide à l’usage des agents de santé communautaire ont déjà été réalisées dans le cadre de notre étude, les futurs coûts d’adaptation devraient être inférieurs. Les méthodes appliquées dans la présente étude offrent un exemple de la façon de chiffrer le coût des adaptations d’interventions dans le futur.

## Resumen

**Objetivo:**

Evaluar el coste de la cocreación de una adaptación de la Guía Internacional para el Seguimiento del Desarrollo Infantil (intervención para su uso por trabajadores comunitarios de salud [TCS ]) en zonas rurales de Guatemala e India.

**Métodos:**

Se desarrollaron instrumentos de encuesta para estimar los costes, desde las perspectivas del sistema sanitario y de la sociedad, de la adaptación de la guía con socios locales y expertos internacionales en ambos emplazamientos. Se incluyeron los costes de recursos humanos, tecnologías de la información y apoyo de infraestructura y logístico. Para mejorar la transparencia y la comparabilidad de las estimaciones de costes, se describieron los recursos necesarios para la adaptación, como el tiempo y la experiencia del personal, así como la cantidad, la función y el tiempo de uso de los recursos utilizados.

**Resultados:**

La adaptación de la guía requirió 14 meses en India y 18 meses en Guatemala durante el periodo 2021-2022. Los costes totales por emplazamiento fueron de 38 174,57 dólares de los Estados Unidos (US$) en Guatemala, US$ 39 287,15 en India y US$ 81 846,59 correspondientes a consultores internacionales. Los consultores internacionales representaron aproximadamente la mitad de los costes, mientras que los emplazamientos de Guatemala e India representaron cada uno alrededor de una cuarta parte. Los recursos humanos constituyeron el principal componente de los costes de adaptación en ambos emplazamientos (90,1%; 34 398,20/38 174,57 en Guatemala y 94,8%; 37 262,74/39 287,15 en India), seguidos del apoyo a la infraestructura y de las tecnologías de la información.

**Conclusión:**

Dado que la mayoría de las etapas necesarias para adaptar la guía para su uso por trabajadores comunitarios de salud ya se han llevado a cabo en este estudio, se espera que los costes de adaptación futuros sean menores. Los métodos utilizados en este estudio proporcionan un ejemplo de cómo estimar los costes de adaptaciones de intervenciones en el futuro.

## ملخص

**الغرض:**

تقييم تكلفة المشاركة في إنشاء نسخة مُعدّلة من الدليل الدولي لرصد نمو الطفل (تدخل لاستخدامها بواسطة العاملين في مجال الصحة المجتمعية (CHW) في المناطق الريفية في غواتيمالا والهند.

**الطريقة:**

قمنا بتطوير أدوات للمسح لرصد تكاليف تكييف الدليل، من منظور النظام الصحي والمنظور المجتمعي، بالتعاون مع شركاء محليين وخبراء دوليين في الموقعين. وقمنا بتضمين تكاليف الموارد البشرية، وتكنولوجيا المعلومات، والبنية التحتية، والدعم اللوجستي. ولتحسين شفافية تقديرات التكلفة وقابلية مقارنتها، قمنا بإعداد تقارير عن الموارد المطلوبة للتكييف، مثل وقت وخبرة الموارد البشرية، وكمية الموارد المستخدمة ووظيفتها ومدة استخدامها.

**النتائج:**

استغرق تكييف الدليل 14 شهرًا في الهند و18 شهرًا في غواتيمالا خلال الفترة من 2021 إلى 2022. وبلغت التكاليف الإجمالية حسب الموقع 38174.57 دولارًا أمريكيًا في غواتيمالا، و39287.15 دولارًا أمريكيًا في الهند، و81846.59 دولارًا أمريكيًا للاستشاريين الدوليين. شكل الاستشاريون الدوليون حوالي نصف التكاليف، بينما شكلت تكاليف كل من غواتيمالا والهند حوالي ربع التكاليف. كانت الموارد البشرية المساهم الأكبر في تكاليف التكييف في كلا الموقعين (%90.1؛ 34398.20/38174.57 في غواتيمالا، و%94.8؛ 37262.74/39287.15 في الهند)، يليها دعم البنية التحتية وتكنولوجيا المعلومات.

**الاستنتاج:**

نظرًا لأن معظم الخطوات المطلوبة لتكييف الدليل لاستخدامه بواسطة العاملين في مجال الصحة المجتمعية، قد تم إنجازها في دراستنا، فإننا نتوقع انخفاض تكاليف التكييف المستقبلية. تقدم الأساليب المستخدمة في هذه الدراسة مثالًا على كيفية حساب تكاليف تكييف التدخلات في المستقبل.

## 摘要

**目的:**

旨在评估共同制定《儿童发展监测国际指南》改编版本【供危地马拉和印度农村地区的社区卫生工作者 (CHW) 采取干预措施时使用】所需成本。

**方法:**

我们开发了调查工具，以从卫生系统和社会的角度计算在这两个国家聘请当地合作伙伴和国际专家改编指南所需成本。我们将人力资源成本、信息技术成本以及基础设施和后勤支助成本全部计算在内。为了提升成本估计的透明度和可比性，我们报告了改编指南所需资源（例如人力资源方面的时间投入和专门知识投入）以及所用资源的数量、功能和使用时间。

**结果:**

在 2021-2022 年期间，印度和危地马拉分别历时 14 个月和 18 个月完成了指南改编工作。按现场划分算得的总成本分别为 38,174.57 美元（危地马拉）、39287.15 美元（印度）和 81846.59 美元（聘请国际顾问的花费）。聘请国际顾问的花费约占成本的一半，而危地马拉和印度两个现场的支出分别约占成本的四分之一。在两国改编成本中，占比最大的都是人力资源支出（在危地马拉占 90.1%，即 34398.20/38174.57；在印度占 94.8%，即 37262.74/39287.15），其次是在基础设施支助和信息技术方面的支出。

**结论:**

由于我们现已在开展研究的过程中完成了大部分指南改编（以供 CHW 使用）相关必要步骤，我们预计未来的改编成本将会降低。我们在本次研究中所用方法为未来如何计算干预措施改编成本提供了范例。

## Резюме

**Цель:**

Оценить затраты на совместное создание адаптированной версии Международного руководства по мониторингу развития детей (предназначенного для использования общинными медико-санитарными работниками (ОМСР) в сельских районах Гватемалы и Индии).

**Методы:**

Авторы разработали опросники, чтобы определить затраты (с точки зрения системы здравоохранения и общества) на адаптацию руководства с привлечением местных партнеров и международных экспертов в двух странах. В расчет включались затраты на кадровые ресурсы, информационные технологии, инфраструктуру и материально-техническую поддержку. Чтобы повысить прозрачность и обеспечить сравнимость оценок затрат, авторы представили данные о требуемых для адаптации ресурсах, таких как время, квалификация кадровых ресурсов, количество, функциональное назначение и время использования каждого из ресурсов.

**Результаты:**

Адаптация руководства заняла 14 месяцев в Индии и 18 месяцев в Гватемале на протяжении 2021–2022 годов. Общие затраты составили 38 174,57 доллара США для Гватемалы, 39 287,15 доллара США для Индии и 81 846,59 доллара США на международных консультантов. Почти половина расходов пришлась на международных консультантов, а расходы на местах в Гватемале и Индии составили примерно по четверти от общей суммы. Наибольшая доля затрат в обеих странах пришлась на оплату кадровых ресурсов (90,1%, 34 398,20/38 174,57 доллара США в Гватемале и 94,8%, 37 262,74/39 287,15 доллара США в Индии), за которыми следовали инфраструктурная поддержка и информационные технологии.

**Вывод:**

Поскольку большая часть шагов для адаптации руководства к использованию ОМСР была выполнена в ходе описываемого исследования, авторы полагают, что впредь затраты на адаптацию будут ниже. Методы, использованные в данном исследовании, служат примером того, каким образом оценивать затраты на адаптацию мер в будущем.

## Introduction

More than 40% of children younger than 5 years in low- and middle-income countries are at risk of not reaching their developmental potential due to poverty, undernutrition and other factors.^1^^,^^2^ This situation has implications globally, including lost human capital and increased costs to the health and education sectors.^3^

To address this challenge, early childhood development interventions that are scalable are needed. However, these interventions often require highly trained clinical staff, such as primary care physicians or others with advanced training in child development.^4^ Such an approach is not scalable in many low- and middle-income countries due to staffing constraints and is a major barrier to bringing care to children at risk of poor development.

A promising strategy is to task-shift and integrate early childhood development interventions into existing community health worker (CHW) programmes.^5^ Task-shifting to CHWs has many potential benefits, including better reach and cost−effectiveness.^6^ However, CHWs differ markedly from medical professionals in their educational background, literacy level and skills, and how they are perceived within their communities. Therefore, task-shifting early childhood development interventions must include an adaptation process where existing tools, training materials and procedures are modified so that they are appropriate for use by CHWs in their local contexts. Adapting evidence-based interventions for a better fit in new contexts is an established practice and can be more efficient than creating new interventions for every context.^7^

Published reports frequently lack sufficient detail on the adaptation process; those reports that do have described varying degrees of engagement with local partners.^8^^–^^10^ A co-creating approach that involves local partners in adaptation provides many benefits compared with conventional adaptation procedures. This approach ensures user friendliness, cultural sensitivity, fidelity to the original version of the tool and local ownership for internationally developed tools. Knowing the costs of a co-creating adaptation will allow policy-makers and other stakeholders to evaluate the financial feasibility of adapting tools and interventions with local partner involvement.

To fill this knowledge gap, we assessed the cost of a co-creating adaptation of the International Guide for Monitoring Child Development (hereafter called the guide).^11^ This guide is a comprehensive early childhood development package that has been rated among the best available developmental monitoring tools for use with children in low- and middle-income countries.^12^ The guide was originally designed for use by physicians or professionals with training in early childhood development. In 2021–2022, we collaborated with experts, local partners, implementers, CHWs and caregivers to adapt both the guide’s training package and intervention for use by CHWs in two settings.^13^ The adapted guide is currently being evaluated in a cluster randomized controlled hybrid type 1 trial in rural areas of Guatemala and India.^14^ Our current study aims to provide necessary cost information for the adaptation of the guide and to present an example of the procedures required for costing adaptations to interventions.

## Methods

We prepared this manuscript according to Consolidated Health Economic Evaluation Reporting Standards guidelines (online repository).^15^^,^^16^

### Study setting

The adaptation was conducted in multiple departments of the central highlands region of Guatemala and Sevagram, Wardha, a rural district in Maharashtra State in India. Further details are presented in Box 1.

Box 1 **Description of the study settings during the adaptation of the International Guide for Monitoring Child Development, Guatemala and India, 2021–2022**

Guatemala is an upper-middle income country and India a lower-middle income country. The Guatemalan central highlands population is predominately Indigenous Maya with most living below the national poverty line.^17^^,^^18^ In Maharashtra, India, the population is predominately Marathi-speaking with one in six people living below the poverty line.^19^ Both sites represent poor rural areas in their countries and have a high prevalence of mortality and stunting in children younger than 5 years: the Indian site has 31 deaths per 1000 live births with 35% stunted, and the Guatemalan site has 23 deaths per 1000 live births with 46% stunted.^20^ Furthermore, both countries have a high estimated prevalence of children at risk of suboptimal development (70% and 84%, respectively).^2^ In Guatemala, the local partner of the project was Maya Health Alliance, a primary care organization working with private-sector CHWs to provide child and maternal care to rural families of Indigenous Maya ethnicity. In India, the partner was Mahatma Gandhi Institute of Medical Sciences, which works closely with public sector Anganwadi workers in the state of Maharashtra. Anganwadi workers are frontline health workers within the Integrated Child Development Services programme.

### Adaptation of the guide

**Fig. 1:**
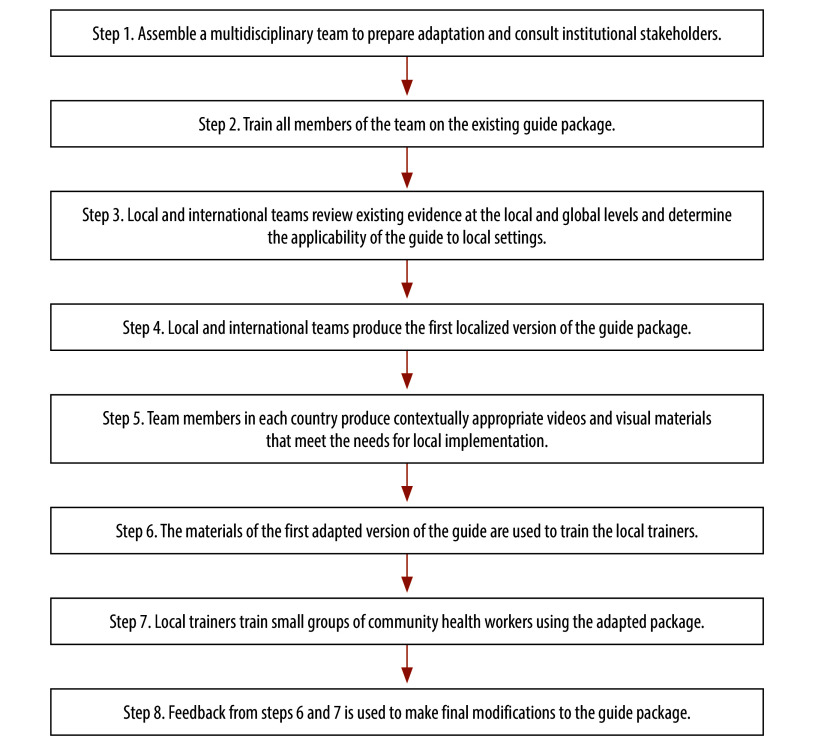
Eight steps used to adapt the International Guide for Monitoring Child Development for use by community health workers, India and Guatemala, 2021–2022

The objective of the guide is to promote optimal early childhood development by partnering with families to support each child individually. The guide is based on family-centred care principles and can be used with all children aged between 0 and 3.5 years (online repository).^16^ The intervention involves interviewing the primary caregiver using 10 open-ended questions. The first seven questions evaluate the child’s function in seven developmental domains, while the last three ask about risk factors and facilitators for the child’s development. The interviewer and caregiver problem-solve together to address identified concerns and risk factors for poor development. In addition, the interviewer provides the caregiver and child individualized developmental support and referrals if needed. Visits are either monthly or quarterly depending on if the child has a risk factor or a developmental delay. The guide has been standardized in four low- and middle-income countries and is currently used in multiple countries across the globe. In low- and middle-income countries, trained health workers such as primary care physicians and nurses mostly deliver the guide.^21^ In our study, we adapted the guide package so tasks could be shifted to CHWs in rural Guatemala and India. The distinctive feature of this adaptation method was to have the active contribution of local partners, including local clinical staff, CHWs, CHW trainers and caregivers. An overview of the adaptation process is shown in Fig. 1.

To establish a common understanding, all local team members in India and Guatemala were initially trained in the pre-existing guide. Then, the teams and the experts reviewed all international and local training and implementation materials and determined the suitability of the intervention components for each local context. Based on the reviewed evidence, the international experts drafted an adapted version of the guide and further revised it based on feedback from the local teams. For example, previously, the guide was used with a digital application (app) that guides the user through the steps of the intervention. We found that CHWs in the Indian and Guatemalan sites were not comfortable using the app and there were also logistical issues for implementing partners. In response to this challenge, we developed a pictorial job aid called the Flipchart, which is a printed booklet that replaces the digital app. In the original guide, the frequency of visits is left to the judgement of the clinician. In the adapted version, we created a simple algorithm to determine the frequency of visits. Children without developmental delays or risk factors receive visits every 3 months. Children with a risk factor or developmental delay receive visits every month. Local team members from Guatemala and India then created country-specific video and photographic materials for the newly adapted guide package. The adapted package was piloted in the two local settings by training local CHWs who then implemented the guide with caregivers. The package was further revised to deal with challenges and issues that emerged during the pilot. Adaptations occurred throughout these steps with back-and-forth communication between the local country teams and the international experts.

The adaptation took 14 months (from October 2021 to December 2022) in India and 18 months (from June 2021 to December 2022) in Guatemala.

### Costing the survey instrument

We developed survey instruments to capture the adaptation costs. In line with previous studies, the surveys used the bottom-up approach recommended by the World Health Organization (WHO), which collects cost data for each personnel member involved and items used in the adaptation.^22^^–^^24^ We designed survey instruments to capture the costs incurred at the health-system level, which were structured around the WHO framework for health systems. We collected cost data from three areas: human resources, which included all personnel involved in the adaptation of the guide, the role and responsibility of the personnel, time spent, and the related salary and benefits; information technology used for the adaptation, which included the quantity and unit price of each item (e.g. mobile telephones and camera) taking depreciation into account; and infrastructure support, which included office space, utilities, furniture and telephone bills.^25^ Taking a societal perspective, we documented the costs of travel time, food and lodging, volunteers' time and in-kind support or donations made to the project. Survey instruments are available in the online repository.^16^ Although we used the same survey instruments to collect cost data in all areas (Guatemala, India and international experts), some subcategories were not relevant to all.

### Collection of cost data 

In both sites, a member of the research team collected cost data with support from the finance and administration staff of local partners and the international experts. A senior costing expert supported the local teams.

Cost data mainly came from four sources: (i) the finance and administration team of the local partners; (ii) the research team; (iii) reported by involved personnel; and (iv) local markets. For example, we collected information on salary and benefits of the personnel from the respective administrative and finance staff of local partners or the research or consultation team. Information on the full-time equivalent of staff committed to adaptation was either from the staff’s self-reported time spent or from the project manager. For items used in the adaptation, local finance teams mainly provided cost information. For example, the finance team provided the costs of travel, lodging and buying laptops through account ledgers and receipts. When using existing items for adaptation (e.g. mobile telephones), we decided the life span of various line items and the proportion of time used for adaptation after discussion with the project staff and local study investigators. We also collected the rental price of the item in the local market. Cost teams categorized the expenses and entered the data under the appropriate category in Excel (Microsoft, Redmond, United States of America). To ensure the data quality, members of the local teams reviewed and verified the costs of items with the records and ledger whenever available to assure minimal errors in data entry.

In both Guatemala and India, we retrospectively collected the cost data from June 2021 until July 2022. From August 2022 to December 2022, we collected the data concurrently or prospectively.

### Cost estimation

We measured costs from a health-system and societal perspective and included opportunity costs that represent both monetary costs and the costs of time or lost potential. Costs from a societal perspective include nonmedical costs such as participants’ travel time, volunteer time and food.

#### Human resources

The costs of human resources include the costs of all people involved in the adaptation. We estimated the costs by multiplying the monthly salary of the personnel by the monthly time spent on adaptation or by their salary as dictated in the grant. In both sites, CHWs participated in adaptation activities by testing training materials. In Guatemala, although CHWs were volunteers, we calculated the costs of their time by using the local minimum wage. In India, CHWs received small incentives, which we included in our analysis. We gave caregivers and children who participated in the adaptation food bags and toys as compensation, the cost of which we included in the costs. We also included the cost of travel and lodging necessary for adaptation in this category. As international consultants provided guidance to both sites, we analysed the costs incurred for them separately from other staff.

#### Information technology

We calculated information technology costs based on the receipts for the items bought for the adaptation. For the existing items used in the adaptation, such as electronics, we used two methods to estimate the costs. With items for which we could find a rental price in the local market of the country, we calculated the costs by multiplying the rental price of the item by its quantity. If unavailable, we followed previous studies and obtained the purchasing receipts and made assumptions about their life span.^24^ We then adopted straight-line depreciation with zero salvage value assumed, and generated the annual value of the item by dividing the purchase costs by its life span. We calculated the costs of the item used for adaptation by multiplying the annual value of the item by number of years used for adaptation.

#### Logistics and infrastructure

Logistic and infrastructure support includes costs for office space, utilities, office supplies and office furniture. We calculated costs for office space and utilities by multiplying the actual monthly rent being paid by the project by the proportion of time used for the adaptation and the number of months spent in adaptation. During enforced lockdown during the coronavirus disease 2019 pandemic, some adaptation activities were performed at home. However, we still included the infrastructure costs as if staff worked in the office. We calculated the costs of using new or existing items in the same way as for the costs of information technology.

#### Additional contextual information

Reporting United States dollar (US$) values of the adaptation is not informative to interested policy-makers and implementers in other settings, because the salary of a position or the unit price of an item can vary widely across settings. To address this issue and improve data quality, transparency and comparability, we followed suggestions from a previous study that itemized costs with additional supplementary information.^24^ For example, when reporting the costs of human resources, we also presented number of personnel, their titles and their roles and responsibilities, and time efforts. This information will enable interested policy-makers in other countries to estimate the costs using their local prices.

We used three types of 2022 price in reporting: 2022 local currencies in the two countries, US$ and international purchasing power parity (PPP). To generate and report the cost estimates in local currencies in 2022, we used gross domestic product deflators (Guatemala: 1.063721; India: 1.058971; base year: 2022) in the two countries to convert the 2021 costs into the costs in 2022 prices.^26^ Based on the costs at the 2022 price in each country, we generated the costs in 2022 international PPP for each country using the conversion rates between their currency and international PPP (1 international PPP = 3.19 quetzales, Q, in Guatemala; 1 international PPP = 20.49 Indian rupees, ₹, in India).^27^ We also generated the costs in 2022 US$ using exchange rates between the local currency and US$ in 2022 in each country (US$ 1 = Q7.75; US$ 1 = ₹78.611).

## Results

Table 1 shows the overall costs of adapting the guide by site. The overall cost was US$ 159 308.31 (US$ 38 174.57 in Guatemala for 18 months, US$ 39 287.15 in India for 14 months and US$ 81 846.59 for international consultants). Human resources was the largest contributor to total costs accounting in all three areas: 90.1% (34 398.20/38 174.57) in Guatemala, 94.8% in India (37 262.74/39 287.15) and 95.9% (78 516.53/81 846.59) for the international consultants. Infrastructure and logistics support were the second largest contributor: 8.4% (3213.17/38 174.57) in Guatemala, 3.3% (1297.35/39 287.15) in India and 3.3% (2684.54/81 846.59) for international consultants. Information technology cost the least: 1.5% (563.20/38 174.57) in Guatemala, 1.9% (727.06/39 287.15) in India and 0.8% (645.52/81 846.59) for international consultants.

**Table 1:** Overall costs of adaptation of the International Guide for Monitoring Child Development, Guatemala and India, 2021–2022

Country and cost category	Total costs (US$)	Total costs (international PPP)	% of total costs
**Guatemala**			
Human resources	34 398.20	83 569.28	90.1
Infrastructure	3 213.17	7 806.29	8.4
Information technology	563.20	1 368.27	1.5
Total	38 174.57	92 743.84	100.0
**India**			
Human resources	37 262.74	142 960.53	94.8
Infrastructure	1297.35	4 977.34	3.3
Information technology	727.06	2 789.42	1.9
Total	39 287.15	150 727.29	100.0
**International consultants for the two countries**			
Human resources	78 516.53	NA	95.9
Infrastructure	2 684.54	NA	3.3
Information technology	645.52	NA	0.8
Total	81 846.59	NA	100.0

NA: not applicable; PPP: purchasing power parity; US$: United States dollar.

Table 2 gives the costs of human resources for Guatemala, India and the international consultants. A wide range of roles were needed in the adaptation, including local clinical staff and local and international researchers. Local staff participated in a variety of activities, including translating documents, collecting photographs, training to become guide trainers, conducting the pilot study, designing study documents and coordinating field activities. International staff provided expertise on the guide training package as well as guidance and support on adapting the materials to the Guatemalan and Indian contexts. Out of the total costs, Guatemala accounted for 24.0% (38174.57/159 308.31) of the costs, India for 24.7% (39287.15/159 308.31) and the international consultants for 51.4% (81 846.59/159 308.31). In Guatemala, 42.0% (14 437.91/34 398.20) of the human resources costs were for three trainers who conducted several other functions while 51.9% (17 492.13/34 398.20) were for one supervisor. In India more personnel were involved who often had narrower functions including: one adaptation coordinator (27.4% (10 208.56/37 262.74) of total human resource cost), three trainers (29.7%; 11 084.59/37 262.74), an office assistant (3.2%; 1181.57/37 262.74), an artist (2.8%; 1042.52/37 262.74), a translator (3.0%; 1122.12/37 262.74), a document editor (0.3%; 105.10/37 262.74), five Anganwadi workers (0.1%; 50.88/37 262.74) and three supervisors (26.7%; 9943.58/37 262.74).

**Table 2:** Itemized costs for human resources used in the adaptation of the International Guide for Monitoring Child Development, Guatemala and India, 2021–2022

Role/title (no. of personnel)	Function	Duration (days)^a^	Cost, US$^b^	Costs, international PPP^c^	Costs in local currency^d^	% of total costs
**Guatemala**						
*Human resources directly involved in adaptation and development*						
Trainer (3)	Training to become guide user and then trainer; training CHWs for pilot study; reviewing, translating and creating intervention documents; developing audio visual material	285.0	14 437.91	35 076.42	111 893.78	42.0
*Human resources for supervising*						
Supervisor (1)	Training Guatemalan staff members to become guide users, then trainers; supervising adaptation	5% full time equivalent over 18 months	17 492.13	42 496.55	135 564.00	50.9
*Other expenses related to human resources*						
Travel for adaptation activities (field visits for photographs, videos and pilot training)	NA	NA	1 499.71	3 643.50	11 622.76	4.4
Food	NA	NA	76.19	185.10	590.47	0.2
Food bags for caregivers and children participating in pilot training	NA	NA	360.00	874.61	2 790.00	1.0

Volunteer support CHWs (5)	Trained to become guide users in pilot project	5	523.26	1 293.10	4 125.00	1.5
Total	NA	NA	34 398.20	83 569.28	266 586.01	100.0
**India**						
*Human resources directly involved in adaptation and development*						
Adaptation coordinator (1)	Training staff to become guide user and then trainer; designing training materials; producing audiovisual material; creating animation videos; coordinating field and adaptation activities	331	10 208.56	39 165.68	802 504.82	27.4
Trainer (3)	Training staff to become guide user and then trainer; providing input for the local adaptation; field activities; collecting audiovisual materials	812	11 084.59	42 526.62	871 370.36	29.7
Office assistant (1)	Field activities, including collecting photographs and videos of local context, resource mapping for services related to developmental delays in children	230	1 181.57	4 533.17	92 884.60	3.2
Artist (1)	Creating animations to be used in the guide package; designing basic pictorial rules and guide handouts	40	1 042.52	3 999.70	81 953.80	2.8
Translator (1)	Translating documents	30	1 122.12	4 305.08	88 211.00	3.0
Document editor (1)	Editing translated documents	15	105.10	403.22	8 262.00	0.3
Anganwadi workers (5)	Coordinating home visits and field activities for adaptations	40	50.88	195.22	4 000.00	0.1
*Human resources for supervising*						
Supervisor (3)	Supervising adaptation; reviewing adaptation materials; meeting with stakeholders to implement pilot training for adaptation	76	9 943.58	38 149.07	781 674.38	26.7
*Other expenses related to human resources *						
Travel for adaptation activities	NA	NA	2 060.78	7 906.30	162 000.00	5.5
Food provided during pilot training	NA	NA	305.30	1 171.30	24 000.00	0.8
Food bags and toys for caregivers and children participating in pilot training	NA	NA	157.74	605.17	12 400.00	0.4
Total	NA	NA	37 262.74	142 960.53	2 929 260.96	100.0
**International consultants**						
*Human resources involved in adaptation and development*						
Expert in early child development and the guide (1)	Leading the preparation and adaptation of materials for two countries; providing online training for trainers including watching guide administration videos; supporting pilot training with adapted packages; finalizing adapted packages	60.1	40 006.17	NA	NA	51.0
Expert in early child development and the guide (1)	Supporting the preparation and adaptation of the training material; providing online support for trainings for two countries	39.5	15 041.92	NA	NA	19.2
Expert in early child development and the guide (1)	Supervising and guiding the development and adaptation of materials	14 months at 20% full-time equivalent	11 460.27	NA	NA	14.6
Project manager and senior trainer (2)	Reviewing training package and providing feedback; supporting guide trainers including checking fidelity	14 months at 30% full-time equivalent	12 008.17	NA	NA	15.3
Total	NA	NA	78 516.53	NA	NA	100.0

CHW: community health worker; Guide: International Guide for Monitoring Child Development; ₹: Indian rupee; NA: not applicable; PPP: purchasing power parity; Q: quetzal; US$: United States dollar.

^a^ 1 day = 8 hours.

^b^ 1 US$ = Q7.75 and ₹78.611 in 2022.

^c^ 1 international PPP = Q3.19 and ₹20.49 in 2022.

^d^ The local currency in Guatemala is quetzal and in India rupee.

Table 3 shows the itemized costs of the information technology that was used in the adaptation of the guide. These costs mainly consist of using devices to adapt the training materials for the previous guide to the new contexts. Computers accounted for most of the costs in the two countries: 75.7% (426.49/563.20) in Guatemala and 59.3% (431.26/727.06) in India. For international consultants, 37.0% (238.93/645.52) of the costs were for computers. Telephones accounted for 17.7% (99.61/563.20) of the costs in Guatemala, 34.9% (253.97/727.06) in India and 50.7% (327.31/645.52) for international consultants.

**Table 3:** Itemized costs for information technology used in the adaptation of the International Guide for Monitoring Child Development, Guatemala and India, 2021–2022

Item	Quantity	Function	Life span, years	Cost, US$^a^	Costs, international PPP^b^	Costs in local currency^c^	% of total costs
**Guatemala**							
Laptop computer	4	Creating, revising and translating documents; use to attend virtual meetings; communication	3	426.49	1036.15	3 305.32	75.7
Printer	1	Printing materials related to adaptation	3	22.49	54.63	174.28	4.0
Mobile telephone	4	Communication; internet	3	99.61	241.99	771.95	17.7
Projector	1	Projecting presentations during pilot training	3	7.00	17.01	54.25	1.2
Sound equipment	1	Speakers and microphone used during pilot training of community health workers	3	7.61	18.49	58.98	1.4
Total	NA	NA	NA	563.20	1368.27	4 364.78	100.0
**India**							
Laptop computer	1	Creating, revising and translating documents; use to attend virtual meetings; communication	4	170.95	655.87	13 438.70	23.5
Mobile telephone	6	Communication; internet	2	253.97	974.38	19 964.98	34.9
Tablet computer	3	Collecting local photographs and videos for module development	2	147.14	564.50	11 566.66	20.2
Desktop computer	2	Preparing animation videos and designing pictorials; maintaining office documents and finances	5	113.17	434.19	8 896.55	15.6
Printer	1	Printing materials related to adaptation	5	21.04	80.70	1 653.64	2.9
Camera	1	Capturing photographs and videos for package adaptation	4	18.32	70.28	1 439.99	2.5
Television	1	Projecting presentations during pilot training of community health workers	10	1.67	6.42	131.58	0.2
Mango Animate Software	1	Creating animation videos	10	0.53	2.05	42.03	0.1
Sound equipment	1	Speakers and microphone used during pilot training of community health workers	5	0.27	1.02	21.00	0.0
Total	NA	NA	NA	727.06	2789.42	57 155.13	100.0
**International consultants**							
Computers	5	Creating, revising, translating and reviewing documents; use to attend virtual meetings	Not available	238.93	NA	NA	37.0
Mobile telephone	5	Communication	Not available	327.31	NA	NA	50.7
Microsoft Office	5	Creating and editing adaptation material	Not available	79.28	NA	NA	12.3
Total	NA	NA	NA	645.52	NA	NA	100.0

₹: Indian rupee; NA: not applicable; PPP: purchasing power parity; Q: quetzal; US$: United States dollar.

^a^ 1 US$ = Q7.75 and ₹78.611 in 2022.

^b^ 1 international PPP = Q3.19 and ₹20.49 in 2022.

^c^ The local currency in Guatemala is quetzal and in India rupee.

Table 4 gives the costs for infrastructure support. Most of infrastructure support costs were for office space and utilities: 82.7% (2657.80/3213.17) in Guatemala, 66.9% (867.90/1297.35) in India and 98.0% (2629.53/2684.54) for the international consultants.

**Table 4:** Itemized costs for infrastructure support used in the adaptation of the International Guide for Monitoring Child Development, Guatemala and India, 2021–2022

Item	Costs, US$^a^	Costs, international PPP^b^	Costs in local currency^c^	% of total costs
**Guatemala**				
Office space and utilities	2657.80	6457.03	20 597.94	82.7
Furniture and existing office supplies	371.37	902.24	2 878.15	11.6
New office supplies	184.00	447.02	1 426.00	5.7
Total	3213.17	7806.29	24 902.09	100.0
**India**				
Office space and utilities	867.90	3329.74	68 226.39	66.9
New office supplies	412.21	1581.47	32 404.37	31.8
Furniture and existing office supplies	17.24	66.13	1 354.92	1.3
Total	1297.35	4977.34	101 985.68	100.0
**International consultants**				
Office space and utilities	2629.53	NA	NA	98.0
New office supplies	45.92	NA	NA	1.7
Furniture and existing office supplies	9.09	NA	NA	0.3
Total	2684.54	NA	NA	100.0

₹: Indian rupee; NA: not applicable; PPP: purchasing power parity; Q: quetzal; US$: United States dollar.

^a^ 1 US$ = Q7.75 and ₹78.611 in 2022.

^b^ 1 international PPP = Q3.19 and ₹20.49 in 2022.

^c^ The local currency in Guatemala is quetzal and in India rupee.

## Discussion

This study estimated the costs of a co-creating adaptation of the guide for use by CHWs as part of a cluster-randomized controlled hybrid type 1 trial of the guide in rural India and Guatemala.^14^ International consultants accounted for over half of the total adaptation cost, while the Guatemala and India sites each accounted for under a quarter of the costs. Human resources made up ≥ 90% of the total costs in all sites, indicating that rigorous adaptations are human resource-intensive but do not necessarily require substantial investments in equipment and infrastructure.

Although the adaptation was done in two different settings, the civil sector in Guatemala and the public sector in India,^28^ the overall cost and proportional contributions were similar for each country. The costs of the international consultants, who assisted the adaptation for the two countries separately, were the largest contributor to the total cost. These costs are expected to be lower for future adaptations of the guide in a new setting, because the adapted guide package has now been developed for users with low education levels and no prior experience in early childhood development.^13^

While cost–effectiveness studies on early childhood development interventions are common, literature is scarce on the cost of adapting such interventions, making it difficult to compare our work to other studies. We found no published studies on the costs of adapting early childhood development interventions. A study in India found that the cost of developing a digital training programme for treating depression by CHWs was US$ 208 814.^24^ Although the proportional contribution of human resources to the overall cost was 61.1% (127 628/208 814) less than our adaptation of the guide (≥ 90.0%), it was still the greatest contributor to the costs. The cost of information technology was higher in the referenced study (33.1% (69 070/208 814) versus < 2.0%), driven by the costs of developing digital tools.

Our study has important strengths. First, we undertook the cost estimation in these two countries with different social, political and economic contexts. The findings on the resources required and their costs in the two countries are a useful indicator for others interested in adapting the guide in additional countries. Second, we grouped our costs based on the WHO health system framework, and used standardized survey instruments and checklists which allow for comparisons across different interventions and settings. Third, we included itemized costs, such as the responsibilities of the personnel and the functions of the items assessed. Including additional information such as item function and personnel responsibilities permits people in other settings to determine what items and personnel are necessary for their specific context. Fourth, we have published our participatory co-creating adaptation method elsewhere, which complements this costing analysis.^13^ Our future research will measure the costs of delivering the adapted guide in the two settings. Details on how to implement the guide can be found in the published protocol.^14^ In addition, we will undertake a cost–effectiveness analysis of using the guide to improve early childhood development using the Bayley Scales for Infant Development and the Home Observation Measurement of the Environment scale.

Our study has some limitations. First, the estimation of costs depends on the salary, wage and market prices in the local areas. The absolute value of the costs may fluctuate when large variations exist in these prices across regions of a country or countries. Therefore, information about the amount of time spent and quantity of items required in the adaptation, generated from our study, would be more comparable across the settings and informative to the budget for others interested in adapting the guide. Second, we did not document the participating caregivers’ time or convert it into a monetary value. Instead, we provided food and toys as compensation, which may have different monetary value than their invested time. Third, when costing existing items that were used in the adaptation, we used straight-line depreciation assuming zero salvage, in line with previous studies. The estimated cost could change depending on the depreciation method chosen. For example, if we used straight-line depreciation with positive salvage value, the cost of the used item could be lower. If we used a double-declining balance depreciation method and the item was in its first year, the cost of the item could be higher. Given that most of the costs were for human resources, the cost difference due to using different depreciation methods would likely not be substantial. Finally, cost data from the first year were collected retrospectively which may introduce recall bias.

Recent publications have emphasized the high economic costs of not addressing early childhood development and the relatively low cost of providing services.^3^^,^^29^ Scientific evidence also supports investments in early childhood development programmes which have high benefit–cost ratios and rates of return.^3^^,^^29^^,^^30^ Viewed in this context, the costs incurred while adapting early childhood development programmes, which is the first step to implementation, can be regarded as potential investments in human capital. Our study fills in an important knowledge gap in the assessment of the cost of adapting an early childhood development intervention. Since many steps required to adapt the guide for use by CHWs are now done, we expect future adaptation costs to be lower. 
